# How do alignment programs perform on sequencing data with varying qualities and from repetitive regions?

**DOI:** 10.1186/1756-0381-5-6

**Published:** 2012-06-18

**Authors:** Xiaoqing Yu, Kishore Guda, Joseph Willis, Martina Veigl, Zhenghe Wang, Sanford Markowitz, Mark D Adams, Shuying Sun

**Affiliations:** 1 Department of Epidemiology and Biostatistics, Case Western Reserve University, Cleveland, OH, 44106, USA; 2 Case Comprehensive Cancer Center, Case Western Reserve University, Cleveland, OH, 44106, USA; 3 Department of Medicine, Case Western Reserve University, Cleveland, OH, 44106, USA; 4 Department of Pathology, Case Western Reserve University, Cleveland, OH, 44106, USA; 5 J. Craig Venter Institute, 10355 Science Center Dr, San Diego, CA, 92121, USA

**Keywords:** Next generation sequencing, Alignment, Sequencing quality, SOAP2, Bowtie, BWA, Novoalign

## Abstract

**Background:**

Next-generation sequencing technologies generate a significant number of short reads that are utilized to address a variety of biological questions. However, quite often, sequencing reads tend to have low quality at the 3’ end and are generated from the repetitive regions of a genome. It is unclear how different alignment programs perform under these different cases. In order to investigate this question, we use both real data and simulated data with the above issues to evaluate the performance of four commonly used algorithms: SOAP2, Bowtie, BWA, and Novoalign.

**Methods:**

The performance of different alignment algorithms are measured in terms of concordance between any pair of aligners (for real sequencing data without known truth) and the accuracy of simulated read alignment.

**Results:**

Our results show that, for sequencing data with reads that have relatively good quality or that have had low quality bases trimmed off, all four alignment programs perform similarly. We have also demonstrated that trimming off low quality ends markedly increases the number of aligned reads and improves the consistency among different aligners as well, especially for low quality data. However, Novoalign is more sensitive to the improvement of data quality. Trimming off low quality ends significantly increases the concordance between Novoalign and other aligners. As for aligning reads from repetitive regions, our simulation data show that reads from repetitive regions tend to be aligned incorrectly, and suppressing reads with multiple hits can improve alignment accuracy.

**Conclusions:**

This study provides a systematic comparison of commonly used alignment algorithms in the context of sequencing data with varying qualities and from repetitive regions. Our approach can be applied to different sequencing data sets generated from different platforms. It can also be utilized to study the performance of other alignment programs.

## Background

The great demand for efficient, inexpensive, and accurate sequencing has driven the development of high-throughput sequencing technologies from automated Sanger sequencing to next-generation sequencing (NGS) over the past several years. Currently, NGS technologies are capable of producing low-cost data on a giga base-pair scale in a single run, which usually includes millions of sequencing reads. This ability makes the NGS technology a powerful platform for various biological applications, such as genetic variant detection by whole-genome or target region resequencing, mRNA and miRNA profiling, whole transcriptome sequencing, ChIP-seq, RIP-seq and DNA methylation studies. The first step of nearly all these applications is to align sequencing reads onto a reference genome. Thus, in order to obtain any further genetic information from sequencing data, the requirement of fast and accurate alignment tools has to be a priority [1].

In parallel with the rapid growth of new sequencing technologies, many alignment programs [2-20] have been developed, including MAQ, Novoalign (http://www.novocraft.com), SOAP, Bowtie, and BWA. Among all these five aligners, MAQ is the only one that indexes the reads, while all other aligners build indexes on a reference genome. In terms of the indexing algorithms they adopt, MAQ and Novoalign are two alignment programs that build an index with a hash table. To identify inexact matches in short-read alignments, MAQ uses a split strategy while Novoalign adopts an alignment scoring system based on the Needleman-Wunsch algorithm [21]. SOAP2 employs a similar split strategy as MAQ in identification of inexact matches. Instead of using a hash table, SOAP2 adopts the FM-index algorithm [22] to build an index, which greatly reduces the alignment time for substrings with multiple identical copies. Bowtie and BWA are two other alignment programs developed based on the FM-index method that uses a backtracking strategy to search for inexact matches. These programs serve as relatively efficient and accurate tools in aligning large number of reads, and greatly extend the scale and resolution of sequencing technology applications.

New challenges for alignments have arisen from applying sequencing technologies to address different biological questions. For example, how do reads with various sequencing qualities affect alignment results? How do they deal with the reads that can be mapped to multiple locations on a reference genome? In order to answer these questions, we select four commonly used aligners (SOAP2, Bowtie, BWA, and Novoalign), and conduct a systematic analysis to evaluate the performance of these programs. First, we review and compare the algorithms these alignment programs employ as well as their advantages with respect to the major options they provide. Then, we use two sets of real Illumina sequencing data and two sets of simulated data to study how different alignment programs perform on sequencing data with varying quality and from repetitive regions. The performance is measured in terms of 1) concordance between any pair of the aligners, and 2) accuracy in simulated read alignment. We have demonstrated that, for sequencing data with reads that have relatively good quality or have had the low quality bases trimmed off, all four alignment programs perform similarly. Furthermore, we show that trimming off low quality ends markedly increases the number of aligned reads and improves the consistency among different aligners as well, especially for low quality data. However, Novoalign is more sensitive to the improvement of data quality. As for aligning reads from repetitive regions, our simulated data show that reads from repetitive regions tend to be aligned incorrectly, and suppressing reads with multiple hits can improve alignment accuracy.

## Methods

### Reviewing the features of alignment programs

Hash table and suffix tree are two major indexing algorithms that current alignment programs use. Hash table indexing, which was first introduced into the field of alignment by BLAST [23], keeps the positions of k-mer query subsequence as keys, and then searches for the exact match of the keys in reference sequences. It consumes less space since it builds an index for positions of sequences instead of the sequences themselves. Among different suffix tree algorithms, FM-index is based on the Burrows-Wheeler transforms (BWT) [24]. BWT is a reversible permutation of characters in a text. It transforms the original character string into a more compressed format, where the same characters are placed side by side as a cluster, rather than in a scatter pattern. Out of the four alignment programs we are interested in, Novoalign adopts a hash table algorithm, while SOAP2, Bowtie, and BWA adopt the FM-index (Table 1).

**Table 1:** Algorithm of four aligners: SOAP2, Bowtie, BWA, and Novoalign

	**SOAP2**	**Bowtie**	**BWA**	**Novoalign**
	(2.20)*	**(0.12.3)**	**(0.5.8 C)**	**(2.07.00)**
Indexing	FM-index	FM-index	FM-index	Hash table
Inexact match	Split read	Quality-aware backtracking	Backtracking	Alignment scoring

*version of the program.

To find inexact matches, alignment programs allow a certain number of mismatches using different strategies (Table 1). SOAP2 uses a split-read strategy to allow at most two mismatches. A read will be split into three fragments, such that the mismatches can exist in, at most, two of the three fragments at the same time. Bowtie uses a backtracking strategy to perform a depth-first search through the entire space, which stops until the first alignment that satisfies specific criterion is found [15]. Similar to Bowtie, BWA also adopts a backtracking strategy to search for inexact matches. However, the search in BWA is bounded by the lower limit of number of mismatches in the reads. With this limit better estimated, BWA is able to define a smaller search space, and thus make the algorithm more efficient [16]. Moreover, BWA provides a mapping quality score for each read to indicate the Phred-scaled probability of the alignment being incorrect. This mapping quality score incorporates base qualities, number of mismatches, and the repeat structure. The higher the mapping quality score, the more accurate an alignment is. A zero will be assigned if a read is aligned to at least two locations with equal probabilities. On the other hand, Novoalign first finds candidate alignment positions from the reference genome for each read, and calculates alignment scores for these positions using the Needleman-Wunsch algorithm, based on base qualities, the existence of gap, and ambiguous codes (Ns). This alignment score is ­10log_10_(*q*), where *q* represents the probability of observing the read sequence given a specific alignment location. This score corresponds to the parameter setting “-t” at the command line when running Novoalign which finds the best alignment with the lower score and any other alignments with similar scores. Because of this alignment-score-based search algorithm, users cannot define the number of allowed mismatches in each alignment, but they can set up a threshold of alignment scores.

We also summarize the major options that the four alignment programs provide (Table 2). SOAP2, Bowtie, BWA, and Novoalign all allow pair-end alignments, enable the identification of the best alignment, and incorporate certain ways of trimming low quality bases (Table 2). There are some characteristics unique to certain aligners. For example, in BWA, the maximum number of allowed mismatches is sensitive to the length of reads. If less than 4% of *m*-long reads with 2% uniform base error rate have more than *k* mismatches, then the maximum number of allowed mismatches in these reads is set to be *k*. Thus, for our simulated data with 50-bp-long reads, *k* = 3. For the real NGS data with 68-bp-long reads, *k* = 4 (Table 2). This number may vary depending on the length of reads after trimming. Unlike the other programs, Novoalign does not allow the users to define the number of allowed mismatches. However, this parameter can be set indirectly by defining the threshold of the alignment score. In practice, setting the threshold at ‘-t 60’ will be approximately equivalent to allowing two mismatches at high quality base positions and maybe one mismatch at a low quality position.

**Table 2:** Available options in SOAP2, Bowtie, BWA, and Novoalign

	**SOAP2 (2.20)**	**Bowtie (0.12.3)**	**BWA (0.5.8 C)**	**Novoalign (2.07.00)**
Mismatch allowed	exactly 0,1,2	max in seed, 0-3 max in read	up to k*	up to 8 or more in single end;
Alignments reported per read	random/all/none	up to any	up to any	random/all/none/
Gap alignment	1-3 bp gap	unavailable	available	up to 7 bp
Pair-end reads	available	available	available	available
Best alignment	minimal number of mismatch	minimal number of mismatch	minimal number of mismatch	highest alignment score
Trim bases	3’ end	3’ and 5’ end	available	3’ end**

*Given a read of length *m*, less than 4% of *m*-long reads with 2% uniform base error rate may have more than *k* mismatches. For *m* = 15-37 bp, *k* = 2; for *m* = 38-64 bp, *k* = 3; for *m* = 64-92 bp, *k* = 4; for *m* = 92-123 bp, *k* = 5; for *m* = 124-156 bp, *k* = 6.

**only available for single-end reads.

### Alignment performance evaluation

#### Data sets

**Figure 1:**
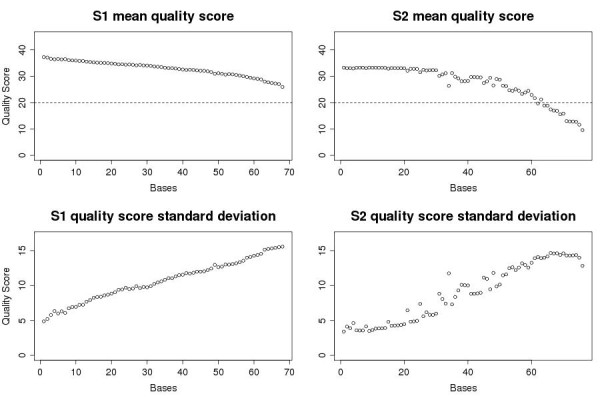
**Mean quality score and standard deviation for each base in S1 and S2 data sets.** Quality score is assessed in Illumina FASTQ format.

In order to examine how the four selected alignment programs (i.e., SOAP2, Bowtie, BWA, and Novoalign) perform on real sequencing data with varying quality, we use two single-end Illumina sequencing data sets (S1 and S2). S1 and S2 are sequenced from human colon cancer samples. For each of these two samples, about 3000 exons selected from cancer related genes are captured and sequenced by the Illumina sequencer, with 7,406,247 and 5,398,566 68-bp-long single-end reads generated respectively. We process the reads with ShortRead package inside of Bioconductor (http://www.bioconductor.org) to evaluate the bases quality. The plot of base qualities suggests that S1 has an overall better quality than S2. For example, S2 has more low quality bases at the 3’ end. In particular, the last 10 bases have average quality score less than 20 (Figure 1).

In order to examine how the four selected alignment programs perform on sequencing data obtained from repetitive regions, we simulate two sets of data from human genome 18: 1) 138771 50-bp-long reads are generated from about 3000 exon regions from which the real data sets S1 and S2 are generated, and these 3000 exon regions do not have many repetitive regions; 2) 55018 50-bp-long reads are generated from 218 CpG islands. These 218 CpG islands are selected from 28226 CpG islands along the whole genome, by the criteria that each chosen CpG island must have at least 25% repetitive bases, and these repetitive bases must be at least 50 bp in length. Note that the purpose of this article is not to simulate reads from all repetitive regions in a genome. It is difficult to precisely define repetitive regions in a genome. Therefore, we simply choose to use some CpG islands that have some level of repetitive regions.

In order to mimic the situation that one or two single bases are mismatches due to pure sequencing errors or true novel single nucleotide variants (SNV), we design the following four scenarios in our simulation data:

1) randomly set one mismatch for each read and let all bases have high quality;

2) randomly set two mismatches for each read and let all bases have high quality;

3) randomly set one low quality mismatch for each read and let all other bases have high quality;

4) randomly set two low quality mismatches for each read and let all other bases have high quality.

1) and 2) are the cases that the mismatches are likely due to the existence of novel SNVs. 3) and 4) are the cases that the mismatches are likely due to pure sequencing errors. Low quality bases are the ones with Phred quality score ranging from 5-15; high quality bases are the ones with Phred quality score ranging from 30-40. Phred quality score is defined as -10*log_10_(*p*), where *p* is the base-calling error probability.

#### Trimming, alignment, and evaluation

In order to evaluate how different alignment programs perform in sequencing reads with low quality ends, we use the four alignment algorithms to map S1 and S2 before and after trimming off the low quality bases using BRAT trim [18], respectively. In particular, we set the parameters of BRAT as trimming from both the 5’ and 3’ ends until reaching a base with quality score higher than 20, and allowing at most two Ns in each read. The length of the trimmed reads is at least 24 bases, and the majority of them are larger than 50 bases. We then perform alignments against the human genome 18 on trimmed and non-trimmed S1 and S2 data using SOAP2, Bowtie [15], BWA [13], and Novoalign (http://www.novocraft.com), respectively. For the purpose of this study, we set the parameters in all four alignment programs as follows: 1) At most two mismatches are allowed in SOAP2, Bowtie, and BWA for each alignment. Due to the different alignment searching algorithm that Novoalign uses, we set the parameter t at 60 to allow approximately up to two mismatches, and then choose the alignment reads with no more than two mismatches using the NM tag in the output. 2) Randomly report one alignment for each read, or only report reads with unique alignments. For each alignment result we calculate the percentage of aligned reads. The performance of four alignment programs is measured in terms of concordance between any pair of aligners because no known truth for real sequencing data is available. In particular, for each pair of aligners, aligned reads are assigned into four classes as follows:

Class 1: a read is aligned to the same location by both aligners that we are comparing (e.g., aligner 1 and aligner 2);

Class 2: a read is aligned to different locations by both aligners;

Class 3: a read is only aligned by one of the two aligners (e.g., aligner 1);

Class 4: a read is only aligned by the other aligner in a comparison pair (e.g., aligner 2).

**Figure 2:**
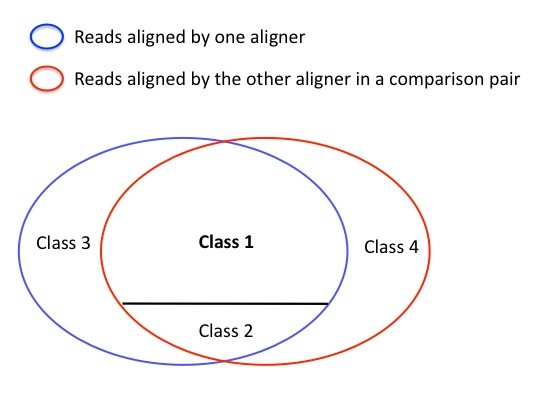
**The four classes to which all reads are assigned during a pair-wise comparison.** Class1 is a group of reads each of which is assigned to the same location by aligners 1 and 2; Class 2 is a group of reads each of which is assigned to a different location by aligner 1 and 2; Class 3 is a group of reads each of which is only aligned by aligner 1; Class 4 is a group of reads each of which is aligned only by aligner 2.

If two alignment algorithms perform similarly, there should be a relatively small number of reads in class 2, 3 and 4 as shown in Figure 2.

In order to evaluate how different alignment algorithms perform on data containing reads generated from regions with more repetitive sequences, we use two simulated data sets. One data set is simulated from the 3000-exon regions that do not have a lot of repetitive bases and the other one is from 218 selected CpG islands that have many repetitive bases. For both simulated data sets, we align these reads using the four selected alignment programs. While aligning these simulated reads, all parameters are set the same as the ones used in real NGS data, except that we allow one mismatch for those data sets with only one mismatch simulated. Because we know the position from which each simulated read is generated, the performance of the four alignment tools is measured in terms of the accuracy of simulated read alignment. We define a true alignment as a situation when a read is aligned back into the same position from which it was generated. In addition, a false alignment is defined as a read that is aligned to other positions rather than the one from which it was generated.

## Results

### Benchmark of aligners

To assess the speed of index building and reads mapping in these four aligners, we use the non-trimmed S1 data, which has 7.4 million 68-bp-long single-end reads. We align these reads using the human genome 18 as a reference, with at most two mismatches allowed and one alignment randomly reported for each read (Table 3). Novoalign is extremely fast (4.02 min) at index building, while the other three take more than one hour to finish the same job (Table 3). As for reads mapping, SOAP2 and Bowtie have a similar number of reads mapped although SOAP2 takes 6 minutes less than Bowtie. BWA maps 76.12% of all reads, which is slightly more than SOAP2 and Bowtie, within 26.4 minutes. Novoalign, on the other hand, is much more time-consuming. It takes 62.9-minute CPU time to align 73.64% of reads in single-end mode.

**Table 3:** Indexing and alignment time of four alignment programs

**Programs**	**Index time (min)**	**Alignment time (min)**	**Reads aligned (%)**
SOAP2 (2.20)	89.50	15.4	75.96
Bowtie (0.12.3)	192.00	21.2	75.71
BWA (0.5.8 C)	101.50	26.4	76.12
Novoalign (2.07.00)	4.02	62.9	74.61

Index is built on the human genome 18 for each aligner. 7.4 million single-end reads are then mapped onto the human genome 18. The read length is 68 bp. At most two mismatches are allowed in all programs, and one alignment is randomly reported for each read. The CPU time in minutes on dual quad-core 2.66Ghz Xeon E5430 processor for index building and alignment processing, as well as percent of mapped reads, are shown in this table.

### Aligners’ performance on sequencing data with different qualities

For the data set S1 that has relatively good quality, all four aligners generally show a good concordance, without trimming off low quality bases. A similar number of reads is aligned by each aligner (Table 4). Over 95% of the reads are assigned into class 1 (i.e., more than 95% of reads are aligned to the same locations by both aligners) when comparing SOAP2, Bowtie, and BWA, pairwise, while Novoalign shows slightly less agreement (84-88%) with the other three aligners (Table 5). However, for the S2 data set that has very low quality bases at many reads, the comparison results are quite different. In non-trimmed data set S2, when Novoalign is compared with any of the other three aligners, less than 50% of reads are assigned to class 1 (i.e., less than 50% of reads are aligned to the same locations by both aligners), but 15% of the reads are assigned to class 2 (i.e., 15% of reads are aligned to different locations by two aligners), and over 30% are assigned to classes 3 or 4 in total (i.e., about 30% of reads are aligned by only one of the two aligners), respectively (Table 6). That means for Novoalign and any other aligner (SOAP2, Bowtie, or BWA), only 50% of all aligned reads are mapped by both of them. This inconsistency of Novoalign’s performance in different data sets might result from the fact that S2 has overall lower quality than the S1 data set (Figure 1). To further investigate the effect of sequencing quality, we trim both S1 and S2 data sets with BRAT trim, and then do alignment using the four aligners.

**Table 4:** Percentage of reads aligned in S1 and S2 data sets by four aligners under different settings

		**S1**		**S2**	
		**w/o trim**	**w/trim**	**w/o trim**	**w/trim**
		**7,406,247**	**7,006,805**	**5,398,566**	**5,193,655**
Randomly report one alignment per read	SOAP2	75.96%	91.45%	42.12%	76.81%
	Bowtie	75.71%	91.36%	41.83%	76.67%
	BWA	76.12%	91.80%	41.94%	76.88%
	Novoalign	73.64%	91.60%	34.50%	76.94%
Suppress reads w/ multiple alignments	SOAP2	71.85%	85.90%	39.75%	71.31%
	Bowtie	68.82%	81.90%	38.89%	68.63%
	BWA	74.40%	84.07%	39.12%	69.75%
	Novoalign	69.67%	86.09%	32.63%	71.63%

**Table 5:** Agreement among aligners in S1 non-trimmed data

	**Comparison pair**	**Class 1**	**Class 2**	**Class 3**	Class 4
Randomly report one alignment per read	SOAP2 vs. Bowtie^1^ (5,626,038)^2^	96.25%	3.41%	0.34%	0.002%
	SOAP2 vs.BWA (5,656,559)	95.72%	3.40%	0.34%	0.54%
	Bowtie vs. BWA (5,637,504)	95.80%	3.66%	0.00002%	0.54%
	SOAP2 vs. Novoalign (5,757,260)	85.13%	7.32%	5.27%	2.28%
	Bowtie vs. Novoalign (5,748,724)	85.18%	7.26%	5.13%	2.47%
	BWA vs. Novoalign (5,835,451)	85.20%	7.24%	5.37%	2.19%
Suppress reads with multiple alignments	SOAP2 vs. Bowtie (5,321,512)	95.78%	0.00002%	4.22%	0.003%
	SOAP2 vs.BWA (5,361,466)	96.50%	0.0005%	2.75%	0.75%
	Bowtie vs. BWA (5,213,871)	97.76%	0.00%	0.0004%	2.24%
	SOAP2 vs. Novoalign (5,447,206)	88.14%	4.27%	5.28%	2.31%
	Bowtie vs. Novoalign (5,432,410)	84.72%	4.08%	5.02%	6.18%
	BWA vs. Novoalign (5,458,788)	85.92%	4.11%	5.48%	4.49%

1. Comparison pair in the format of aligner 1 vs. aligner 2.

2. Total number of reads aligned by either of these two aligners in a comparison pair.

**Table 6:** Agreement among aligners in S2 non-trimmed data

	**Comparison pair**	**Class1**	**Class 2**	**Class 3**	**Class 4**
Randomly report one alignment per read	SOAP2 vs. Bowtie^1^ (2,209,957)^2^	95.69%	3.62%	0.69%	0.003%
	SOAP2 vs.BWA (2,215,397)	95.45%	3.61%	0.69%	0.25%
	Bowtie vs. BWA (2,200,129)	95.37%	3.70%	0.00%	0.25%
	SOAP2 vs. Novoalign (2,436,379)	49.58%	15.40%	25.72%	9.26%
	Bowtie vs. Novoalign (2,424,001)	49.81%	15.38%	25.53%	9.46%
	BWA vs. Novoalign (2,428,458)	49.68%	15.44%	25.48%	9.40%
Suppress reads with multiple alignments	SOAP2 vs. Bowtie (2,085,316)	97.84%	0.00%	2.15%	0.007%
	SOAP2 vs.BWA (2,094,218)	97.57%	0.0008%	1.99%	0.43%
	Bowtie vs. BWA (2,052,464)	99.94%	0.00%	0.0003%	0.59%
	SOAP2 vs. Novoalign (2,303,060)	51.37%	13.36%	25.71%	9.46%
	Bowtie vs. Novoalign (2,283,644)	50.93%	13.35%	25.07%	10.65%
	BWA vs. Novoalign (2,292,171)	50.86%	13.33%	25.35%	10.46%

1. Comparison pair in the format of aligner 1 vs. aligner 2.

2. Total number of reads aligned by either of these two aligners in a comparison pair.

Performing trimming on NGS data not only cuts off the low quality bases from both ends, but also discards poor quality reads, and thus improving reads’ quality markedly. After trimming, 399,442 (5.4%) and 204,911 (3.8%) reads are discarded from S1 and S2 data, respectively. With slightly fewer reads available for alignment, however, the number of aligned reads is increased by 15-17% in the S1 data, and 34-42% in the S2 data, for all four-alignment programs. This apparent difference in the magnitude of increase indicates that trimming has a greater effect on the S2 data set than on the S1 data set. Another interesting observation is that, Novoalign aligns 42% more reads in trimmed S2 than non-trimmed S2, while this increment in the other three aligners is only about 35%, suggesting that data quality improvement has a larger effect on Novoalign.

By trimming off the reads before alignment, we observe a substantial increase in the number of reads that fall into class 1 in all pair-wise comparisons, in both S2 and S1 data sets (Tables 5, 6, 7, and 8). That is, more reads are aligned to the same locations by the comparison pair. This increase indicates an improved concordance among the four aligners. Moreover, trimming appears to have a greater effect on S2, a data set with lower quality, than the S1 data set. In the pair-wise comparisons between Novoalign and any of the other three aligners for S2 data set, the number of reads assigned to the first class increases almost 3-fold, (1.2 million vs. 3.7 million), while the number of reads that are only aligned by the opponents of Novoalign become markedly less (see class 2 of Table 8), compared to non-trimmed alignments (see class 2 of Table 6). On the other hand, in the S1 data set, trimming only improves the agreement between Novoalign and the other three aligners by 8-10% (Tables 5, 7). This differentiation in the magnitude of concordance improvement, along with the fact that performing trimming leads to a more significant improvement in reads’ quality for S2 data set, further indicates that Novoalign is more sensitive towards the changes in sequencing quality.

**Table 7:** Agreement among aligners in S1 trimmed data

	**Comparison pair**	**Class 1**	**Class 2**	**Class 3**	**Class 4**
Randomly report one alignment per read	SOAP2 vs. Bowtie^1^ (6,409,534)^2^	95.89%	3.95%	0.13%	0.03%
	SOAP2 vs.BWA (6,440,873)	95.42%	3.92%	0.13%	0.52%
	Bowtie vs. BWA (6,432,433)	95.30%	4.21%	0.00002%	0.49%
	SOAP2 vs. Novoalign (6,430,033)	94.62%	4.84%	0.13%	0.35%
	Bowtie vs. Novoalign (6,422,084)	94.77%	4.84%	0.07%	0.33%
	BWA vs. Novoalign (6,435,917)	94.83%	4.84%	0.30%	0.05%
Suppress reads with multiple alignments	SOAP2 vs. Bowtie (6,020,802)	95.29%	0.0002%	4.68%	0.003%
	SOAP2 vs.BWA (6,068,512)	96.26%	0.0005%	2.93%	0.81%
	Bowtie vs. BWA (5,890,868)	97.42%	0.00%	0.0004%	2.58%
	SOAP2 vs. Novoalign (6,043,150)	98.47%	0.95%	0.18%	0.40%
	Bowtie vs. Novoalign (6,035,510)	94.11%	0.92%	0.06%	4.92%
	BWA vs. Novoalign (6,066,586)	95.62%	0.92%	0.57%	2.90%

1. Comparison pair in the format of aligner 1 vs. aligner 2.

2. Total number of reads aligned by either of these two aligners in a comparison pair.

**Table 8:** Agreement among aligners in S2 trimmed data

	**Comparison pair**	**Class 1**	**Class 2**	**Class 3**	**Class 4**
Randomly report one alignment per read	SOAP2 vs. Bowtie^1^ (3,890,070)^2^	94.94%	4.84%	0.20%	0.02%
	SOAP2 vs.BWA (3,900,529)	94.69%	4.82%	0.20%	0.29%
	Bowtie vs. BWA (3,892,602)	94.77%	4.96%	0.0002%	0.27%
	SOAP2 vs. Novoalign (3,909,055)	93.84%	5.32%	0.34%	0.50%
	Bowtie vs. Novoalign (3,901,709)	94.50%	5.30%	0.15%	0.50%
	BWA vs. Novoalign (3,908,656)	93.96%	5.30%	0.33%	0.41%
Suppress reads with multiple alignments	SOAP2 vs. Bowtie (3,611,489)	96.20%	0.0002%	3.79%	0.02%
	SOAP2 vs.BWA (3,636,423)	96.42%	0.0007%	2.87%	0.70%
	Bowtie vs. BWA (3,531,986)	98.38%	0.00%	0.0007%	1.62%
	SOAP2 vs. Novoalign (3,638,616)	98.07%	0.54%	0.32%	0.76%
	Bowtie vs. Novoalign(3,631,179)	95.06%	0.52%	0.11%	4.31%
	BWA vs. Novoalign (3,652,782)	97.99%	0.54%	0.70%	3.31%

1. Comparison pair in the format of aligner 1 vs. aligner 2.

2. Total number of reads aligned by either of these two aligners in a comparison pair.

### Aligners’ performance on reads with multiple alignments

To evaluate these aligners in terms of their performance on reads with multiple alignments, we set the alignment parameters in two different ways: (1) randomly report one alignment for each read and (2) only report the read with a unique position (suppress reads that can be aligned to multiple locations). Compared to the former strategy, the latter discards around 4-10% of aligned reads from S1 and 2.5-8% in S2 (see Table 4).

In pair-wise comparisons among all four aligners, we find that in both S1 and S2 data sets, suppressing multiple alignments decreases the number of reads aligned to different positions (class 2) in all comparison pairs, while the number of reads aligned to same positions (class 1) stays the same (Tables 5, 6, 7, 8). Reads with multiple alignments are more likely to be aligned to different locations by different aligners, due to the difference in alignment strategies these aligners employ, as well as the standards of how to randomly choose one alignment to report. Therefore, the number of reads assigned to class 2 during comparison is reduced by suppressing multiple alignments. Next, we will use simulated data to investigate further.

### Aligners’ performance on simulated data

In order to study the four aligners’ performance on reads from repetitive regions, we use the two sets of simulated data as mentioned in the Data Set subsection. One data set is simulated from 3000 exon regions that do not have many repetitive bases. The other data set is from 218 selected CpG islands that have a lot of repetitive bases. In these two simulated data sets, no matter whether the mismatch positions are designed to have high or low quality, all four aligners show a lower false alignment rate in the data set generated from 3000 exon regions (0.7-5%, see Table 9A, B) compared to the data set generated from 218 CpG islands that have more repetitive regions (14-17%, see Table 10A, B). Since the reads from regions with repetitive bases have a much higher probability of being aligned onto multiple locations, we can predict that suppressing multiple hits can help to diminish the false alignments caused by repetitive bases. As expected, the alignment accuracy in CpG island simulation data is substantially improved by suppressing multiple alignments (Table 10A, B).

**Table 9:** Percentage of aligned reads and the false alignment rate for 3000 exon simulation data

**A. Mismatches with high quality (30-40)**						
Mismatch	Settings		SOAP2	Bowtie	BWA	Novoalign
1	Randomly report one alignment	aligned (%)	100	100	100	100
		False alignments (%)	0.76	0.77	0.76	4.83
	Suppress reads w/multiple alignments	aligned (%)	98.69	98.65	98.68	98.69
		False alignments (%)	0	0	0	4.13
2	Randomly report one alignment	aligned (%)	100	100	100	100
		False alignments (%)	0.78	0.78	0.76	8.95
	Suppress reads w/multiple alignments	aligned (%)	98.69	98.68	98.68	98.67
		False alignments (%)	0	0	0	8.26
**B. Mismatches with low quality (5-15)**						
Mismatch	Settings		SOAP2	Bowtie	BWA	Novoalign
1	Randomly report one alignment	aligned (%)	100	100	100	100
		False alignments (%)	0.77	0.75	0.76	3.10
	Suppress reads w/multiple alignments	aligned (%)	98.69	98.65	98.68	98.69
		False alignments (%)	0	0	0	4.13
2	Randomly report one alignment	aligned (%)	100	100	100	100
		False alignments (%)	0.77	0.81	0.76	5.49
	Suppress reads w/multiple alignments	aligned (%)	98.69	98.68	98.68	98.67
		False alignments (%)	0.02	0	0	4.78

**Table 10:** Percentage of aligned reads and the false alignment rate for 218 CpG island simulation data

**A. Mismatches with high quality (30-40)**						
Mismatch	Settings		SOAP2	Bowtie	BWA	Novoalign
1	Randomly report one alignment	aligned (%)	100	100	100	100
		False alignments (%)	13.80	13.84	13.80	17.25
	Suppress reads w/multiple alignments	aligned (%)	84.26	84.26	84.26	84.34
		False alignments (%)	0	0	0.01	4.09
2	Randomly report one alignment	aligned (%)	100	100	100	100
		False alignments (%)	13.90	13.98	13.91	20.77
	Suppress reads w/multiple alignments	aligned (%)	84.39	84.22	84.39	84.23
		False alignments (%)	0.21	0	0.02	8.20
**B. Mismatches with low quality (5-15)**						
Mismatch	Settings		SOAP2	Bowtie	BWA	Novoalign
1	Randomly report one alignment	aligned (%)	100	100	100	100
		False alignments (%)	13.79	13.83	13.80	15.93
	Suppress reads w/multiple alignments	aligned (%)	84.26	84.26	84.26	84.34
		False alignments (%)	0	0	0.001	2.42
2	Randomly report one alignment	aligned (%)	100	100	100	100
		False alignments (%)	13.82	13.86	13.91	17.79
	Suppress reads w/multiple alignments	aligned (%)	84.39	84.22	84.39	84.23
		False alignments (%)	0.21	0	0.02	4.86

By assigning mismatches with high quality, we mimic the true novel variants that are more likely to have better quality. By assigning mismatches with low quality, we mimic the pure sequencing errors. In both cases, SOAP2, Bowtie, and BWA are found to have similar false alignment rates no matter whether the alignment report is randomly reporting one alignment or suppressing reads with multiple hits (Tables 9 and 10). However, Novoalign exhibits higher false alignment rates compared to the other three aligners.

## Discussion

Trimming off the low quality ends of reads improves their quality, and thus improves their alignment results. Although the number of reads available for alignments decreases after trimming, we still observe an increase in the number of successfully aligned reads as well as in the concordance among aligners. S1, with a higher mean and a smaller deviation of base quality score, clearly has better quality than S2 (Figure 1). Thus, it is predictable that trimming has a greater effect on the S2 data set than on the S1 data set, which has been shown by our data analysis. Having a lower quality at the 3’ end is a commonly observed problem in single-end sequencing data, especially in the early version of the Illumina sequencer. By trimming, which only takes a few minutes to process for a data set with several million reads, users can benefit greatly. For example, more information can be extracted from the data since more reads will be aligned after trimming. With the improvement in alignment quality and quantity seen here, we recommend trimming prior to any alignment and downstream analysis, especially for poor quality data.

In the better quality data set S1, Novoalign performs similarly to SOAP2, Bowtie, and BWA, no matter which set of parameters we use. However, in the lower quality data set S2, Novoalign shows patterns that are different from the other three aligners. For example, Novoalign aligns more reads than the others and shows a greater increase in the number of aligned reads after trimming (Table 5). This might be due to the differences in alignment algorithms between Novoalign and the others. As we have shown, in SOAP2, Bowtie, and BWA, the alignment strategy is restrained by the number of mismatches allowed. That means users can specify the number of mismatches they prefer for any alignment process to obtain optimal results for their purpose. Unlike the other three, Novoalign uses an alignment score as a criterion. This alignment score is calculated based upon the base qualities, the existence of gaps, and the ambiguous codes for the entire read. For Novoalign, setting the threshold of the alignment score “-t” at 60 in the command line ensures that only the alignments with an alignment score of no more than 60 are reported, which is approximately equivalent to allowing two mismatches in alignment. However, this is only the case when the quality of reads is within a reasonable range. When applying these aligners to poor quality data sets, such as S2, Novoalign may become more sensitive to the quality and therefore show quite different results as compared to SOAP2, Bowtie, and BWA. After trimming off the low quality ends, the quality of reads has been improved. Thus, the Novoalign results become more similar to the others.

Since the alignment results may be sensitive to the choice of the alignment score threshold, especially for the lower quality data S2, we explore the impact of this parameter “-t” in Novoalign by setting it at different values: default, 60, 70, and 75. For both S1 and S2 data sets, ‘default value’ decreases the concordance of Novoalign with other aligners dramatically; using 70 and 75, the concordance of Novoalign and other aligners is similar as the one using 60. Therefore, we conclude that the pattern of lower concordance of Novoalign with others in a poor quality data set is not due to improper parameter choice.

Other than Novoalign, Bowtie also allows users to have the option of considering the qualities of mismatches. It enables users to set the maximum permitted total of quality values at all mismatched positions throughout the entire alignment (i.e., the “-e” option when setting parameters to run Bowtie). To investigate this parameter setting in Bowtie, we both allow 2 mismatches and set the parameter “–e” at 20, 40, 60, and 80 respectively (data not shown). For our data sets, when the “-e” parameter is set at 40, 60 and 80, there are nearly identical results as compared to the output from only setting the number of allowed mismatches at 2 (i.e., “-v 2”). But setting “–e” at 20 shows severe departures from other three aligners. In our data sets, most reads have moderate to good quality scores. However, setting “–e” at 20 only allows extremely low quality mismatched positions, and therefore, rules out the majority of reads with high quality mismatched positions.

Like trimming off the reads, suppressing multiple alignments also improves the consistency among the three aligners (Table 6). Out of the multiple locations of the reference genome that one read can be aligned onto, only one is true. Even though all aligners can choose one alignment for each read, based on a certain standard, there is no guarantee that the one they choose represents the true location. Thus, eliminating all reads having multiple alignments will help improve the accuracy of alignments and also the consistency among the four aligners. Our analysis resulting from the S1 and S2 data sets supports this conclusion. We design one simulated data set that contains many repetitive bases. By eliminating reads with multiple alignments, the false alignment rate decreases to almost 0 for SOAP2, Bowtie, and BWA, and below 9% for Novoalign (Table 10).

**Figure 3:**
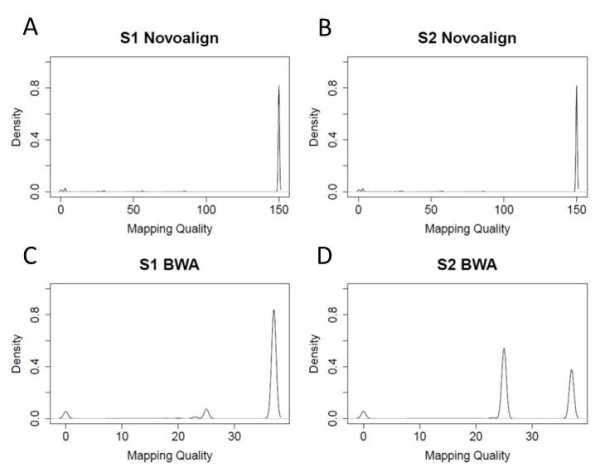
**Mapping quality scores reported in Novoalign and BWA.** Alignment is performed on both the untrimmed S1 and S2 data sets, with one alignment randomly reported for each read.

In addition to the trimming and initial parameter setting of aligners, we also investigate the impact of filtering the alignments based on the mapping quality score provided in the output files of different aligners. Out of the four aligners, BWA and Novoalign both have a mapping quality score reported for each alignment. For BWA, this score is approximately a Phred-scaled probability of the alignment being incorrect, which takes the values of 37, 25, and any value between 23 and 0. In general, a score of 37 means the read is aligned to a unique position with less than 2 mismatches; a score of 25 means the read is aligned to a unique position with 2 mismatches; a score between 23 and 0 means the read is aligned to multiple locations, such that a lower score means that the mapped location is less accurate (based on BWA source code). For Novoalign, the mapping quality score is the probability of the alignment given the read and genome, which ranges from 0 to 150. Higher scores mean better alignment qualities. To explore the effect of quality score filtering, we checked the mapping quality scores in the untrimmed S1 and S2 data with one alignment reported randomly (Figure 3). The distribution of scores shows that both aligners yield alignments with high mapping quality scores. For Novoalign, the majority of reads have a mapping quality score of 150 (Figure 3A and B), which is the upper limit of the score. While for BWA, the majority of reads have a score of 37 or 25 (Figure 3C and D), which means each of them is explicitly aligned to a unique position with 0 to 2 mismatches. A small fraction of reads have scores between 23 and 0. These reads are generally mapped to multiple locations in the reference genome. Therefore, quality score filtering wouldn’t show much impact on the concordance among aligners in the real data sets. In addition, since SOAP2 and Bowtie do not have alignment quality scores in their respective output files, to ensure a relatively fair comparison, no alignment quality filters are used.

As for the mapability of those target regions that we used in our simulation data, we have checked the mapability using the “Duke uniqueness 35 bp” method provided by the UCSC genome browser for the 218 CpG islands and 3000 exon regions. This Duke method reports a mapability score between 0 and 1, with 1 representing a completely unique sequence. A score of 0.5, 0.3, 0.25, or 0 represents that the sequence occurs twice, three times, four times, or more than four times, respectively. For the 218 CpG islands, 80.09% are completely unique, which means all 35-bp sequences within these islands occur only once in the genome; while 19.91% are not completely unique, which means at least one 35-bp sequence within each of these islands occurs more than once in the genome. The median mapability score of all CpG islands is 1 and the mean is 0.9830. For the 3000 exon regions, 95.40% are completely unique and 4.60% are not completely unique. The median mapability score of all regions is 1 and the mean is 0.9930. Generally speaking, the 3000 exon simulation data has better mapabilty than the 218 CpG island data.

There are different ways to evaluate the current available programs. For example, Ruffalo et al. developed a simulation and evaluation suite to compare a few available aligners only using simulated data [25]. In this article, we focus mainly on comparing them from two specific angles (i.e., using real reads with varying qualities and simulated reads from repetitive regions). Thus, there are a few limitations in the article. First, rather than from the whole human genome, both the real data and the simulated data are from part of it. Second, our sequencing data sets are only from the Illumina sequencer. Third, we mainly use single-end sequencing data without considering pair-end data. Fourth, there are many other great alignment algorithms [2,4-10,12,14,19,20] that we did not compare. Although this article has these limitations, our approach is very general, and it can be applied to the pair-end whole genome real and simulated sequencing data as well as data generated from other platforms. It can also be utilized to study the performance of other alignment programs with some minor modifications if necessary.

## Conclusions

In order to study how alignment programs perform on data with varying quality and from repetitive regions, we have evaluated the performances of four commonly used alignment programs—SOAP2, Bowtie, BWA, and Novoalign—on two real NGS data sets and two simulated data sets. Our results show that, for sequencing data with reads that have relatively good quality or have had the low quality bases trimmed off, all four alignment programs perform similarly. We have also demonstrated that trimming off low quality ends markedly increases the number of aligned reads and improves the consistency among different aligners, especially for low quality data. However, Novoalign is more sensitive to the improvement of data quality. Trimming off low quality ends increases the concordance between Novoalign and the others significantly. Therefore, the quality of sequencing data has a great impact on alignment result, and we highly recommend assessing sequencing quality first and then trimming off low quality base if necessary. As for aligning reads from repetitive regions, our simulation data shows that reads from repetitive regions tend to be aligned incorrectly, and suppressing reads with multiple hits can improve alignment accuracy.

## Competing interests

The authors declare no competing interests.

## Authors’ contributions

XY performed all statistical analyses and drafted the manuscript. KG, JW, MV, ZW, SM and MA were involved in the data collection and helped in preparation of the manuscript. SS provided suggestions on the project and revised the manuscript. All authors have read and approved the final document.
